# The influence of anorexia nervosa on oral health and related parameters potentially relevant to orthodontic treatment: a systematic review and meta-analysis

**DOI:** 10.1007/s00784-024-05774-4

**Published:** 2025-01-22

**Authors:** Christian Niederau, Eda Alman, Marta Rizk, Kathrin Becker, Nikolaus Marx, Franziska A. Coenen, Isabel Knaup, Michael Wolf, Rogerio Bastos Craveiro

**Affiliations:** 1 Department of Orthodontics, Dental Clinic, University Hospital RWTH Aachen, Pauwelsstr. 30, 52074 Aachen, Germany; 2 Department of Orthodontics and Dentofacial Orthopaedics, Charité Universitätsmedizin Berlin, Campus CBF, CC 03, Berlin, Germany; 3 Department of Internal Medicine I, University Hospital of the RWTH Aachen, Pauwelsstr. 30, 52074 Aachen, Germany

**Keywords:** Orthodontic therapy, Anorexia nervosa, Oral manifestation, Oral health, Eating disorder, Saliva

## Abstract

**Objectives:**

Information on the oral health of patients with anorexia nervosa remains not satisfactory. The aim of this systematic review is to evaluate oral health parameters in anorexic patients compared to healthy individuals. Furthermore, potential clinical implications for orthodontic treatment are discussed from an orthodontic perspective.

**Materials and methods:**

Electronic databases were searched for case-control and controlled clinical trial studies on dentofacial manifestations in anorexic patients up to 2/2024. Study selection, data extraction and risk of bias assessment was done independently by two authors. Random-effects meta-analyses of mean differences (MDs) or relative risks (RRs) with their 95% confidence intervals (CIs) were conducted, followed by sensitivity analyses.

**Results:**

Eleven out of 573 initially identified studies were included. They involved oral health analyses of general anorexic patients ≥ 12 years (mean age 18.4). The meta-analysis showed that anorexia nervosa was associated with a significantly increased caries experience (DMFT), plaque accumulation and gingival inflammation (BOP). PH and salivary flow rate were significantly altered in patients with anorexia nervosa, although no significant relationship between α-amylase levels and anorexia nervosa was demonstrated.

**Conclusions:**

These data enabled us to formulate modalities for anorexia-specific orthodontic treatments. Based on the results, patients with anorexia nervosa exhibit an increased risk of caries and gingival inflammatory signs.

**Clinical relevance:**

The systematic information on dentofacial manifestations obtained in this study should be considered to better manage the oral health of anorexia patients.

## Introduction

Eating disorders (ED) are, similarly like mental disorders, characterized by disruption of self-perception and by emotional dysregulation. As a result, patients with eating disorders commonly suffer from a significantly lower body weight and malnutrition due to restriction of food and self-induced vomiting [1]. Prevalence of eating disorders has been increasing among young people, especially in Western countries, and in females. An alarming mortality rate of up to 25% hows the importance of prevention and treatment of such diseases [2]. ED have been recently classified by the American Psychiatric Association in Anorexia Nervosa (AN), Bulimia Nervosa (BN) and eating disorders not otherwise specified (EDNOS). Compared to other eating disorders, AN can usually be diagnosed unambiguously on the basis of clinical parameters. Due to the increasing number of patients with anorexia nervosa in the last 10 years, AN started to be a challenge for the treatment of these patients in clinical areas primarily independent of this disorder as such. AN is defined as a strict restriction of food with intense fear of gaining weight, whereas BN is characterized by binge eating and loose of control over eating habits followed by forced vomiting [3]. All these conditions have numerous adverse mental, physical and social effects. Due to malnutrition, ED can lead to additional medical complications [4]. Oral manifestations depend on the duration and frequency of the disorder, the emesis induced, the use of medicines, the diet, and patient´s oral hygiene [5]. Dependent on these factors, the behavioral patterns in different eating disorder types may have various effects on the oral health status. The analysis of general oral health in patients with AN is therefore relevant to all areas of clinical dentistry.

As the peak age of onset of AN is in adolescence [6], orthodontists, as dental specialists providing complex and long-term treatment affecting alveolar bone and periodontal remodeling, are particularly confronted with a variety of oral symptoms affecting the oral mucosa, periodontium, teeth, salivary glands and perioral tissues found in the general oral health of patients with anorexia nervosa [7]. Due to the long duration of orthodontic treatment (OT), usually starting in adolescence, it is crucial for its quality to understand the impact of AN on oral health and on the tissue remodeling processes induced by orthodontic treatment. In addition, maintaining oral hygiene during orthodontic treatment is a critical factor influencing the success of planned orthodontic treatment, as these patients are susceptible to gingival inflammation and enamel demineralization. In severe cases, orthodontic treatments need to be intermitted to prevent teeth from decalcification due to inadequate oral hygiene status. Psychological stress in susceptible individuals has also been associated with an adversely affected outcome of planned orthodontic treatment [7]. There is evidence that orthodontic treatment itself can initiate or stimulate EDs by causing changes in the individual eating behavior, hence triggering the occurrence of anorexia nervosa [8]. Therefore, it is important for orthodontists to recognize AN and in cases of positive oral indicators to refer such patients to a specialist before continuing orthodontic treatment. However, sufficient explanations to what extent anorexia nervosa is a significant risk factor for orthodontic patients are still lacking and it is not clear how orthodontic treatment needs to be adjusted in patients affected by this condition.

The aim of this systematic review is to provide an overview of specific oral manifestations in general patients with anorexia nervosa. The results will be analyzed and discussed in terms of their potential implications for orthodontic treatment, in order to provide a basis for individual treatment planning in patients with anorexia nervosa.

## Materials and methods

The review protocol was registered in PROSPERO (CRD42023412145). This systematic review was structured and conducted according to the preferred reporting items of the PRISMA statement [9]. The PRISMA checklist is provided in supplementary data 2.

### Focused question

The focused question for literature search was structured according to the PECO format [10, 11]: **Population (P)**: Patients < 12 years; **Exposure (E)**: Anorexia nervosa **Comparator (C)**: patients without anorexia nervosa; **Outcome (O)**: Oral health (bleeding on probing, DMFT index, salivary pH, salivary A amylase, salivary flow rate).

### Search strategy

Four electronic databases: PubMed of US National Library, Web of Science, ClinicalTrials.gov and Cochrane were searched without time restriction up to April 2023 to perform a systematic search. A customized search strategy by following key words was implemented:

Search strategy PubMed/Medline and Web of Science:

((“anorexia nervosa“[MeSH Terms] OR (“anorexia“[All Fields] AND “nervosa“[All Fields]) OR “anorexia nervosa“[All Fields])) AND ((„oral manifestation“ OR „oral health“ OR „oral treatment“) OR (caries) OR („white spot”) OR (enamel) OR (dentin) OR (pulpa) OR (“cavity”) OR (erosion) OR (gingiva) OR (gingivitis) OR (periodontitis) OR (mucosa) OR (saliva) OR (gingival recession) OR (“Gingival crevicular fluid”) OR (bone AND (alveolar OR jaw)) OR (maxilla OR maxillary) OR (mandible OR mandibular) OR (orthodontic) OR (orthodontics) OR (orthodontic treatment) OR („loose tooth“) OR („loose teeth“) OR („tooth loss“) OR („teeth loss“)).

Following keywords were searched in combination with „anorexia nervosa“ on ClinicalTrials.gov and Cochrane:

‘’anorexia nervosa’’ and

”oral manifestation” OR “oral health” OR “oral treatment”.

caries OR “white spot” OR enamel OR dentin OR pulpa OR “cavity” OR erosion.

(bone AND (alveolar OR jaw)) OR (maxilla OR maxillary) OR (mandible OR Mandibular).

orthodontic OR orthodontics OR orthodontic treatment.

loose tooth OR loose teeth OR tooth loss OR teeth loss.

### Study selection

Electronic title management was carried out in Endnote 20 (Clarivate Analytics). Initially, two reviewers (E.A. and R.C.) independently searched the electronic databases, followed by a hand search of the relevant article reference lists to collect possible articles, and screened the titles and abstracts for the following inclusion criteria: English language, studies with patients (≥ 12 years), retrospective and prospective case-control and cohort studies investigating oral manifestations, anorexia nervosa patients, females and males, patients: general population (all ethnicities, community dwelling).

In the next step, all full-text articles identified in the first selection were acquired and evaluated according to the following exclusion criteria: other types of eating disorders, comparison to control group not specified, lack of clinical data on oral manifestations, preclinical in vitro or animal studies. Cohen’s kappa coefficient (κ) was calculated to determine the agreement between reviewers.

### Data extraction and method of analysis

Data extraction was performed independently by two authors regarding oral manifestations of anorexia nervosa (BOP, DMFT, plaque index, salivary a-amylase, salivary PH, unstimulated salivary flow rate). Disagreements were resolved by a third person (C.N.). For each outcome, mean measurement, SD and number of patients were extracted.

The mean and standard deviation were obtained in Excel (Microsoft, USA) for continuous outcome, including patient number, age, gender, body-mass-index, soft tissue recessions, bleeding on probing, DMFT index, plaque index, unstimulated salivary flow rate and pH, salivary a amylase.

### Quality assessment of selected studies

The studies were evaluated according to Newcastle Ottawa Quality Assessment Scale for non-randomized studies. The NOS (Newcastle Ottawa Scale) is a validated method for assessing the quality of non-randomised studies, especially case-control studies, and it focuses on three quality parameters (selection, comparability and exposure). The following categories were analyzed: the selection of the study groups; the comparability of the groups; and the ascertainment of either the exposure or outcome of interest for case-control [12]. The evaluation was conducted by two independent reviewers (E.A., R.C.) on the basis of full-text articles.

### Statistical analysis (meta-analysis)

Heterogeneity among the studies and meta-analysis (i.e., weighted mean differences and 95% confidence intervals - CI) for BOP, DMFT, salivary a-amylase, salivary PH, unstimulated salivary flow rate was assessed with RevMan Web, Version 6.5.2 (The Cochrane Collaboration). Available at http://revman.cochrane.org [13]. Meta-analysis was calculated with a random effects model due to the expected heterogeneity of included studies caused by the study design. Based on this, MD, I² and p-values were reported. Grading of the body of evidence was performed according to the GRADE handbook [12]. Case series and case control studies received a low quality of evidence, whereby non-randomized experimental trials were considered as high-evidence, but downgraded to moderate evidence owing lack of randomization/ lack of concealment of allocation.

## Results

### Study selection

**Fig. 1:**
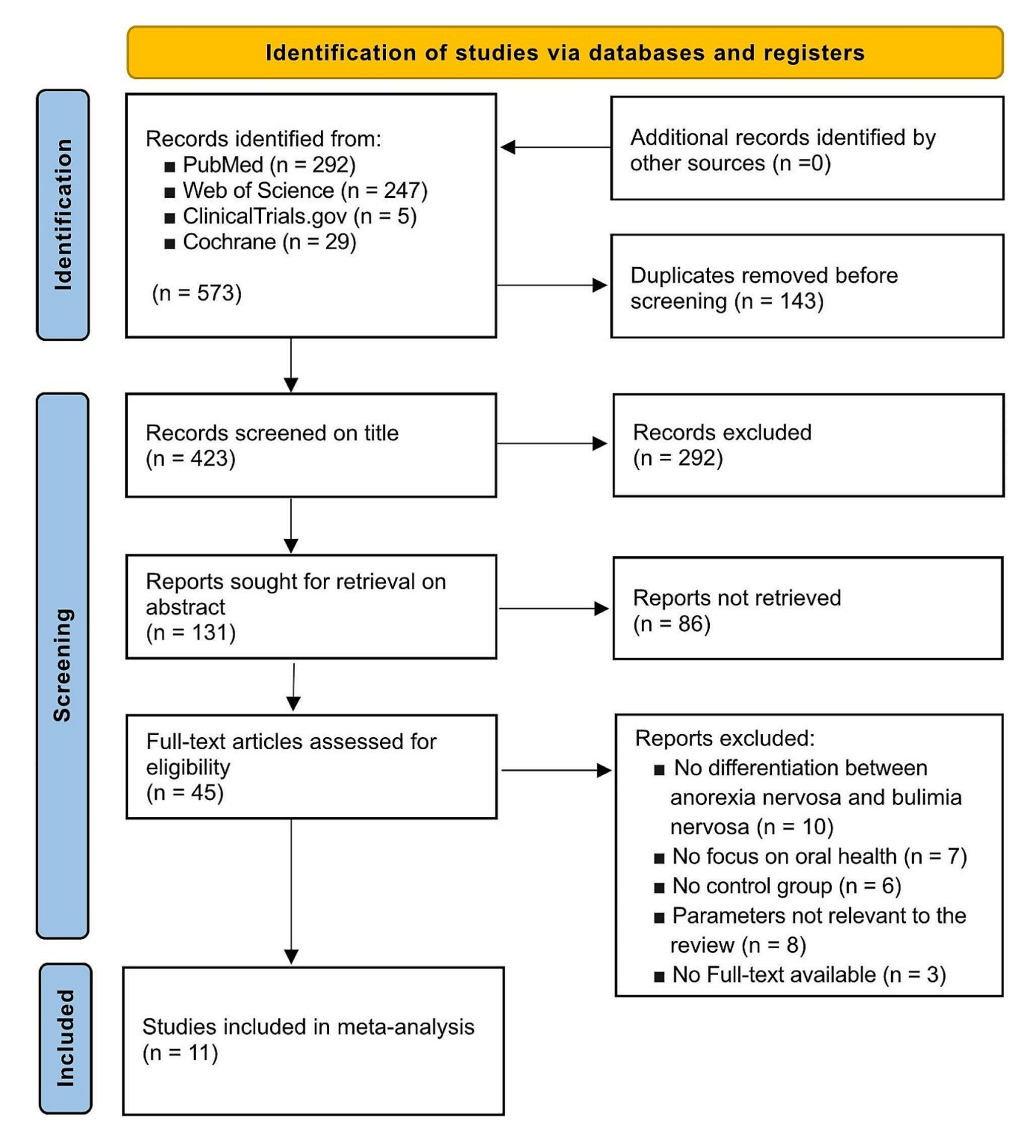
PRISMA flow chart describing the search strategy and study selection. Detailed information about exclusion reasons is shown in supplementary table I

A total of 573 full-text articles were retrieved from four electronic databases (PubMed, Web of Science, Clinical Trials.gov and Cochrane). After removing duplicates, 423 records were screened by title (Cohen’s Kappa = 0.915) and 131 reports were searched by abstract (Cohen’s Kappa = 0.879). Forty-five full text articles were assessed for eligibility and 11 of them were included according to the criteria for the present systematic review (Cohen’s Kappa = 0.886). 11 publications were included in the meta-analysis. (Fig. 1) Detailed information about exclusion reasons is shown in the Supplementary Table I.

### Study characteristics

Publication dates ranged from 1989 to 2022 (Table 1). Literature sources were from Italy (16,67%), France (8,3%), Poland (50%), Germany (8,3%), Australia (8,3%) and USA (8,3%). Twelve articles were included in total, with 345 patients with anorexia nervosa and 12 healthy control groups (Table 1). Most of the studies (50%) used DSM-IV for diagnosis, followed by DMS-III (25%), DSM-IV&V (8,33%) and DSM-V (8,33%). The mean age of the female patients was 18,5 years [13–38, 4]. 75% of the studies were case-control studies, followed by 25% controlled clinical trials. The controlled clinical trials were classified as such by the original authors, although no intervention was performed. The following parameters were assessed in both study groups: periodontal status (REC, BOP), dental status (DMF, erosion), salivary status (pH, flow rate, α-amylase) and oral hygiene (plaque index). Detailed information on the parameters assessed in the included studies is provided under “Oral manifestations/parameters” in Table 1.

**Table 1:** Summary of study characteristics (*n* = 11): DSM- V/IV/III/ III-R: Diagnostic and Statistical Manual of Mental Disorders of Fifth Edition, Fourth Edition; Third Edition; Revision of the Third Edition. DSM is a classification of mental disorders with associated criteria. Data of all studies were retrieved from 100% female patients. Healthy patients of the same gender and age served as control. BOP: Bleeding on probing; BEWE: Basic Erosive Wear Examination; REC: Gingival Recession; PPD: Periodontal pocket depth; DMF: Decayed, Missing, Filled Index; n.a.: not available

Author	Country	Sample size (*N*) / AN patients	Controls (*N*)	BMI AN (SD) (kg/m²)	Diagnostic method	Study design	Oral manifestation/Parameter
Mascitti et al. [4]	Italy	25	25	n.a.	DSM-IV	Case-control study	Periodontal (PA, Gingivitis, BOP) Dental (BEWE) Oral hygiene (Plaque) Oral mucosal disorders
Monteleone et al. [24]	Italy	8	15	16,3 ± 1,2	DSM-IV	Case-control study	Salivary (Cortisollevel, α- amylase level)
Pallier et al. [14]	France	70	36	n.a.	n.a.	Case-control study	Periodontal (REC, PPD, BOP) Dental (DMF, Erosion) Oral Hygiene (Plaque)
Paszynska et al. [19] Dec.	Poland	48	59	13,84 ± 1,34	DSM-IV	Case- control study	Salivary (pH, Flow rate)
Paszynska et al. [19] June	Poland	54	47	n.a.	DSM-IV	Controlled clinical trial	Salivary (Flow rate)
Paszynska et al. [15]	Poland	103	117	16,8 ± 3,6	DSM-V	Case-control study	Periodontal (BOP, DMF, Erosion, Plaque)
Paszynska et al. [39] Nov.	Poland	28	38	n.a.	DSM-IV	Controlled clinical trial	Salivary (Flow rate, α-amlylase)
Paszynska et al. [23]	Poland	20	21	14,09	DSM-IV&V	Case-contol study	Salivary (Flow rate, α-amylase)
Paszynska et al. [22]	Poland	30	30	13,92 ± 1,23	DSM-IV	Controlled clinical trial	Salivary (Flow rate)
Phillip et al. [18]	Germany	11	50	n.a.	DSM-III	Case-control study	Salivary (pH, α-amylase)
Touyz et al. [16]	Australia	15	15	n.a.	DSM-III R	Case control study	Periodontal (REC, PPD, BOP) Dental (DMF) Salivary (pH, flow rate) Oral Hygiene (Plaque)

### Risk of bias in the included studies

The assessment of the risk of bias for each item in all included studies is presented in Table 2. The scale score ranged from 4 to 7 with a mean of 6. One out of eleven studies had a high risk of bias (score < 5). The studies with a high risk of bias were published before 1995. Articles were not excluded from this review using the NOS score (Supplementary material).

**Table 2:** Quality assessment of included studies (*n* = 11) according to NOS Additional information are shown in supplementary material

Non RCT studies	Selection				Comparability	Exposure			Total Quality score
First author	The case definition is adequate with independent validation	Consecutive or obviously representative series of cases	Selection of controls	The control definition is adequate	Comparability of cases and controls on the basis of the design or analyses	Valid exposure assessment	Identical recording of the cases and control	Non-Response-rate available	
Mascitti et al. [4]		*	*	*	**		*		**6**
Monteleone et al. [24]		*		*	**		*		**5**
Pallier et al. [14]	*	*	*	*	**		*		**7**
Paszynska et al. [19] Dec.	*	*	*	*	**		*		**7**
Paszynska et al. [19] June	*	*	*	*	**		*		**7**
Paszynska et al. [15]		*	*	*	**		*		**6**
Paszynska et al. [20] Nov.	*	*	*	*	**		*		**7**
Paszynska et al. [23]	*	*	*	*	**		*		**6**
Paszynska et al. [22]	*	*	*	*	**		*		**6**
Phillip et al. [18]			*	*	*		*		**4**
Touyz et al. [16]			*	*	**		*		**5**

### Periodontal health

The parameters gingival recession and bleeding on probing (BOP) were used to assess information on the periodontal health of patients with AN. In total of all three studies, 168 anorexic participants and 188 control patients were included [14–16]. Mean age of the participants with AN was 25.6 years. BOP was performed as a classical full-mouth examination at 6 sites per tooth using a manual periodontal probe in all three studies. In each study, there was a significant increase in BOP in anorexic patients compared to the control group. Marginal recessions (MR) were measured in millimetres as distance from the dental-enamel junction to the gingival margin. Touyz et al. and Pallier et al. also described a significantly higher proportion of recessions in patients with anorexia nervosa [14, 16].

### BOP – meta-analysis

**Fig. 2:**
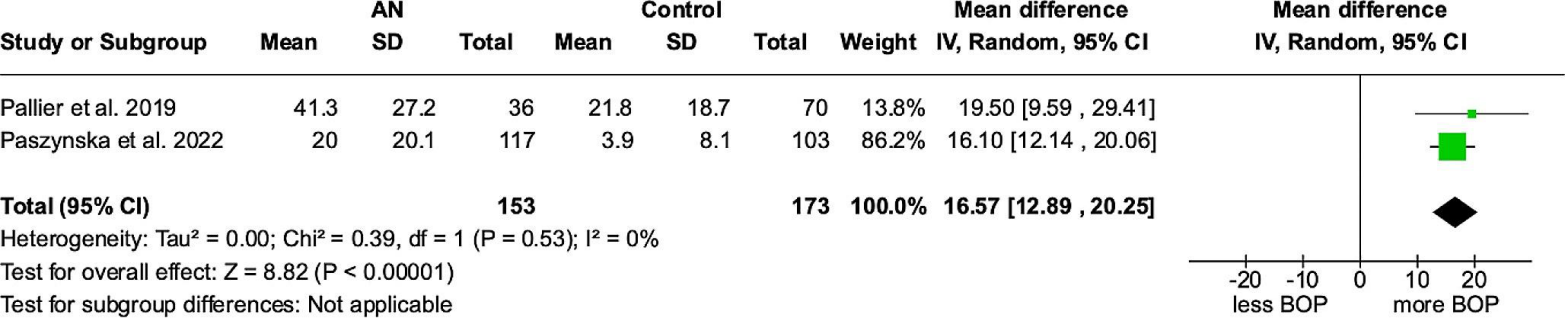
Forest plot for BOP meta-analysis

The meta-analysis for BOP, based on three studies [14–16], revealed significantly increased BOP values in patients with anorexia nervosa with a mean difference (MD) (95% Cl, p) between anorexic patients and control groups of 16.57% (12.89 to 20.25%, *p* < 0.00001) (Fig. 2). The heterogeneity among the analyzed studies was low. (I²=0%)

### Caries experience

Dental examination records included the Decayed, Missing, Filled Teeth (DMFT) score, which measures the number of carious teeth, the number of teeth restored with fillings, and the number of teeth missing due to caries. The DMFT Index is one of the most common methods of measuring the caries experience in dental epidemiology [17]. In total of all four studies 193 anorexic participants and 213 control patients were included [4, 14–16] The mean age of anorexic group was 22.7 years. In 3 out of 4 studies, the DMFT score was significantly higher in people with anorexia. (Table 3)

**Table 3:** Percentage of gingival recession (REC) and bleeding on probing (BOP) in the entire study group. Gingival recession: apical shift of the gingival margin to the cemento-enamel junction (Imber et al. 2021); BOP: Bleeding on probing is an indicator of tissue inflammation. BOP is used to refer to bleeding caused by gentle manipulation of tissue in the gingival sulcus [39].

Reference	Control patients C (*n*)	Anorexia nervosa patients AN (*n*)	Age	Country	REC in % (C/AN)	BOP in % (C/AN)
Pallier et al. [14]	70	36	31.1 ± 7.3	France	0.0 ± 0.1/2.8 ± 3.6 p = < 0.01	21.8 ± 18.7/41.3 ± 27.2 *p* = 0.03
Touyz et al. [16]	15	15	20.1 ± 8.3	Australia	2.0/10.2 p = < 0.001	6.5/16.9 p = < 0.001
Paszynska et al. [15]	103	117	15.1 ± 1.8	Poland		3.9 ± 8.1/20.0 ± 20.1 p = < 0.001

### DMFT – meta-analysis

**Fig. 3:**
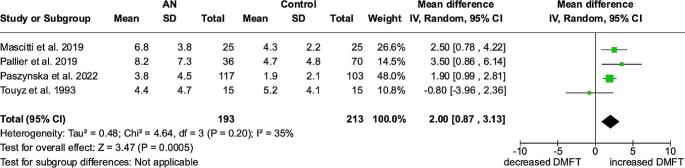
Forest plot for DMFT meta-analysis

Based on four studies [4, 14–16], the MD (95% Cl, p) for DMFT between anorexic patients and control groups amounted to 2.00 (0.87 to 3.13, *p* = 0.0005) with significantly higher values in patients with anorexia nervosa. The heterogeneity among the studies was moderate (I²=35%). (Fig. 3)

### Salivary parameters

Clinical salivary parameters (pH, flow rate and α-amylase content) were analyzed in anorexic patients (mean age 17,2 years). In all included studies, saliva samples were taken from 207 anorexic patients and 264 control patients. Saliva was collected under stimulated and unstimulated conditions. Confounding factors on salivary flow and pH were minimized by testing all participants in the morning and at the same time of the year. In all three studies, salivary pH was significantly lower in anorexic patients than in controls [16, 18, 19]. Stimulated and unstimulated flow rates were also significantly lower in anorexic patients. The results of salivary α-amylase concentration show a high inter-individual variability with no significant evidence. (Table 4)

**Table 4:** Decayed, Missing and Filled Teeth (DMFT) of anorexic patients and control group

Reference	Control patients C (*n*)	Anorexia nervosa patients AN (*n*)	Age	Country	DMFT (c/AN)
Mascitti et al. [4]	25	25	24.5 ± 9.2	Italy	4.3 ± 2.2/6.8 ± 3.8 p = < 0.05
Pallier et al. [14]	70	36	31.1 ± 7.3	France	4.7 ± 4.8/8.2 ± 7.3 p = < 0.01
Paszynska et al. [15]	103	117	15,1 ± 1,8	Poland	1.9 ± 2.1/3.8 ± 4.5 *p* = 0.02
Touyz et al. [16]	15	15	20,1 ± 8,3	Australia	5.2 ± 4.1/4.4 ± 4.7p = NS

### Salivary pH – meta-analysis

**Fig. 4:**
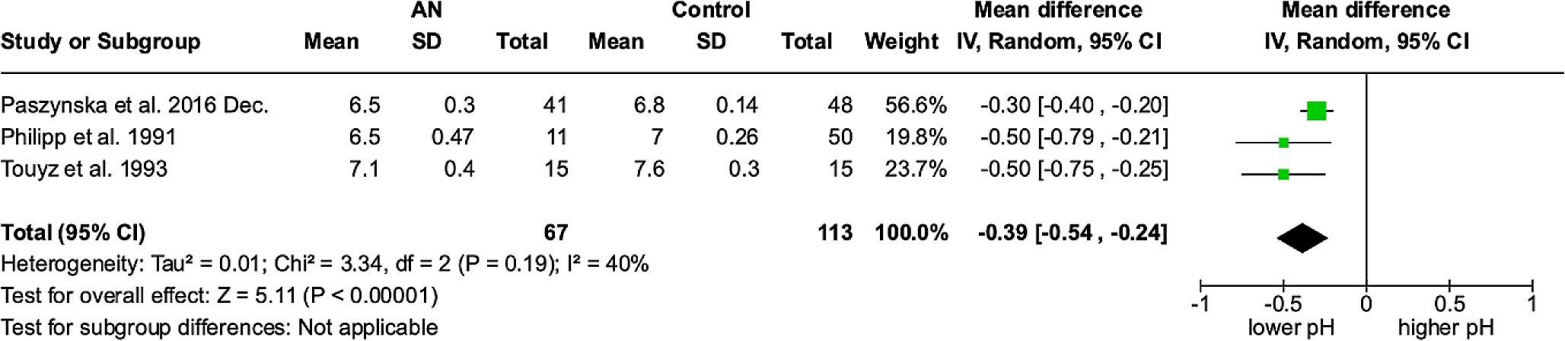
Forest plot for salivary pH meta-analysis

Based on three studies [16, 18, 20] the MD (95% Cl, p) in salivary pH between anorexic patients and control groups resulted in -0,39 (-0,54 to -0,24, *p* < 0.00001) with significantly decreased pH in patients with anorexia nervosa. The heterogeneity was moderate (I² =40%). (Fig. 4)

### Unstimulated salivary flow rate – meta-analysis

**Fig. 5:**
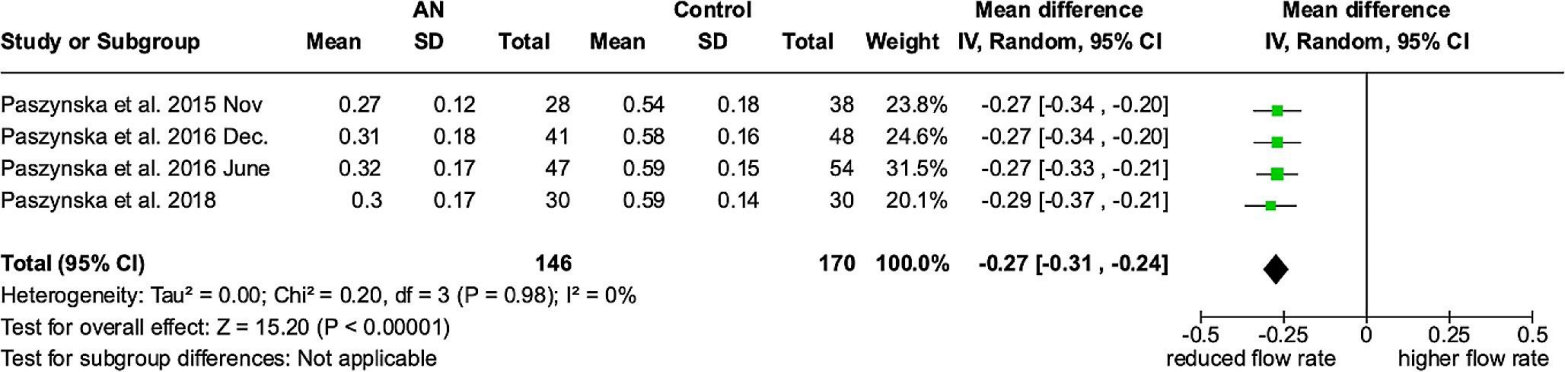
Forest plot for unstimulated salivary flow rate

The meta-analysis for unstimulated salivary flow rate, based on five studies [19–23], showed a MD (95% Cl, p) between anorexic patients and control groups of -0.27 ml/min (-0,31 to -0.24 *p* < 0.00001) with significantly decreased values in patients with anorexia nervosa. The heterogeneity among the studies was low (I²=0%). (Fig. 5)

### α-amylase – meta-analysis

**Fig. 6:**
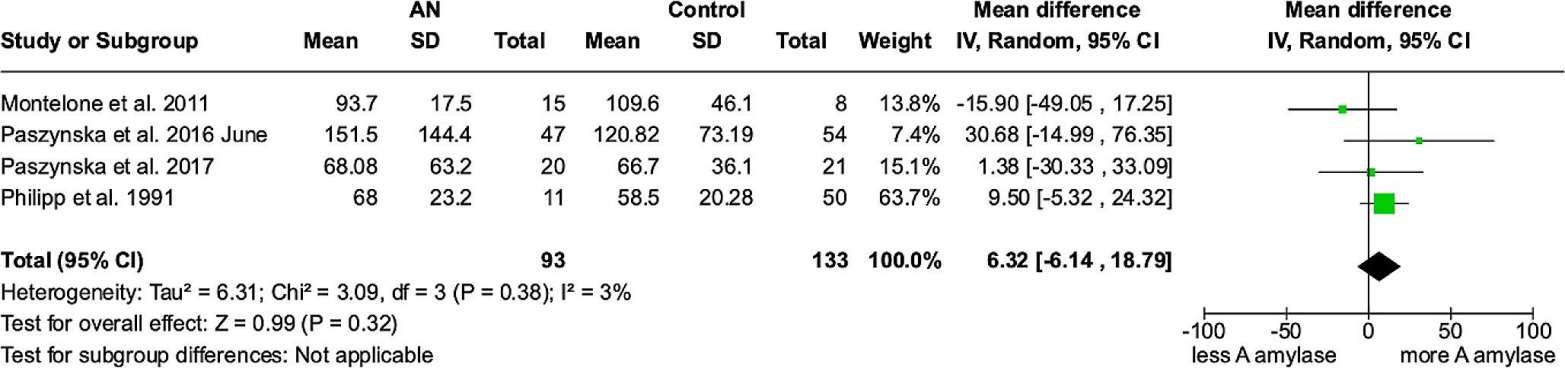
Forest plot α-amylase

Four studies were included for the estimation of MD of in α-amylase [18, 19, 23, 24], resulting in the MD (95% Cl, p) between anorexic and control groups of 6.32 U/I (-6.14 to 18.79 *p* = 0.32), with a low heterogeneity (I²=3%). No difference between the groups was detectable. (Fig. 6)

### Plaque accumulation

For assessing information on the current oral hygiene status, the plaque index was evaluated. In total of three studies [14, 15, 25], 171 anorexic participants and 223 control patients were included. The mean age of anorexia nervosa group was 24.3 years. Pallier et al. & Paszynska er al. used the O’Leary index [14, 15]. This index uses a chart in which each of the 4 surfaces of each tooth is scored. A discloser and an explorer are used to detect the presence of plaque [26]. Milosevic et al. used the Silness and Loe index to measure plaque by recording both soft and mineral deposits on the cervical area of the teeth [25]. A score of 0, 1, 2 and 3 was assigned to each of the four tooth surfaces, i.e., mesiobuccal, midbuccal, distobuccal and midlingual. These scores were then summed and averaged [27]. In 2 out of 3 studies, anorexic patients had a significantly higher prevalence of plaque accumulation (Table 5).

**Table 5:** Clinical and salivary parameters of the anorexic (AN) and control subjects. Salivary pH: Reduced caries activity has been reported in patients with a resting salivary pH of around 7.0 [40]. Salivary flow rate: The state of salivary glands can be monitored by salivary flow rate. The enzyme α-amylase is an activity marker of the central component of the automatic nervous system. It is also known that α-amylase levels are altered in people with eating disorders (Paszynska et al., [19])

Reference	Control patients C (*n*)	Anorexia nervosa patients AN (*n*)	Age	Country	Salivary pH (c/AN)	S (stimulated flow rate) (c/AN) in ml/min	S (unstimulated flow rate) (c/AN) in ml/min	S (α Amylase) (c/AN) in U/l	
Paszynska et al. [19] Dec.	48	41	15 ± 2	Poland	6.8 ± 0.14/6.5 ± 0.3 *p* = 0.0001		0.58 ± 0.16/0.31 ± 0.18 *p* = 0.0001		
Philipp et al. [18]	50	11	22	Germany	7.0 ± 0.26/6.5 ± 0.47 *p* ≤ 0.001			58.5 ± 20.28/68.0 ± 23.2 p = n.a.	
Touyz et al. [16]	15	15	20,1 ± 8,3	Australia	7.6 ± 0.3/ 7.1 ± 0.4 *p* = 0.0001				
Paszynska et al. [20] Nov.	38	28	14 ± 1	Poland		1.57 ± 0.46/1.20 ± 0.34 *p* = 0.005	0.54 ± 0.18/0.27 ± 0.12 *p* = 0.0001		
Paszynska et al. [22]	30	30	15,9 ± 1,6	Poland		1.45 ± 0.27/0.99 ± 0.33 p = < 0.00001	0.59 ± 0.14/ 0.30 ± 0.17 p = < 0.00001		
Paszynska et al. [23]	21	20	15	Poland				66.7 ± 36.1/68.08 ± 63.2 *p* = 0.3185	
Monteleone et al. [24]	8	15	20,2 ± 2,2	Italy				109.6 ± 46.1/ 93.7 ± 17.5 p = n.a.	
Paszynska et al. [19] June	54	47	15,0 ± 2	Poland			0.59 ± 0.15/ 0.32 ± 0.17 *p* = 0.0001	120.82 ± 73.19/151.5 ± 144.4 *p* = 0.8675	

### Grading of the evidence

The majority of studies were case control studies, which were all scored as low evidence, whereas the remaining studies were non-randomized controlled trials and revealed a moderate grading (Table 6).

**Table 6:** Plaque index Pallier et al. [14] & Paszynska er al. [15]: PI was scored using a dichotomised plaque index (O’Leary index). Milosevic et al. 1989: The Silness and Loe index is used to measure the plaque index

Reference	Control patients C (*n*)	Anorexia nervosa patients AN (*n*)	Age	Country	Plaque in % (C/AN)
Pallier et al. [14]	70	36	31.1 ± 7.3	France	53.0 ± 20.4/78.8 ± 19.7 p = < 0.01
Paszynska et al. [15]	103	117	15.1 ± 1.8	Poland	13.7 ± 15.4/43.8 ± 23.4 p = < 0.001
Milosevic et al. [25]	50	18	26.60	USA	72%/84% p = n.a.

## Discussion

Anorexia nervosa tends to start in adolescence, which is also the most common age for orthodontic treatment [5]. Since a variety of oral symptoms affecting the oral mucosa, periodontium, teeth, salivary glands and perioral tissues have been reported in patients with anorexia nervosa [7], it is crucial for orthodontic treatment planning to understand the impact of anorexia nervosa on oral health and possible impairments in tissue remodelling processes during orthodontic tooth movement.

This systematic review evaluates specific oral manifestations of anorexia nervosa in order to identify potential risk factors for orthodontic treatment in these patients. This may provide relevant implications for anorexia-specific orthodontic treatment as well as an important basis for individual treatment plans.

Regarding the oral hygiene parameters of patients with anorexia nervosa, the present meta-analysis showed a significant impairment compared to healthy individuals. Looking at the plaque index scores (Table 5), there is a strong tendency towards increased plaque accumulation in anorexic patients compared to the control group. The presence of biofilm means that the tooth surface is colonized by many pathogenic bacteria, which over time form an organized biofilm that creates an acidic environment [28]. This plaque accumulation can lead to enamel demineralisation, initially manifesting as non-cavitated carious lesions [white spot lesions (WSLs)] and later progressing to cavitated carious lesions. In addition, a shift in the bacterial spectrum can lead to inflammatory signs of the surrounding soft tissues. This is clinically manifested by an increased bleeding tendency during manipulation of the gingiva and the gingival sulcus surrounding the tooth [29]. 

Systematic evaluation of gingival bleeding (BOP) showed a significant rise of BOP in the anorexic group (Fig. 2). This can increase the risk of side effects during orthodontic treatment such as bone loss and tooth loosening. In addition, the incidence of gingival recessions is significantly higher in anorexic patients than in controls (Table 7) [14, 16]. However, the development of marginal recessions is a multifactorial process. Gingival biotype, plaque accumulation and inflammatory signs can be predisposing factors. Although some studies show no significant deterioration in periodontal parameters in ED patients [18, 30], a tendency towards a higher incidence of gingivitis, periodontitis and gingival recession has been presented in the literature [16, 31]. The results of our meta-analysis on BOP support this assumption.

**Table 7:** Summary of findings table

Outcome	No. of participants/ studies	Quality of evidence (GRADE)	Anticipated mean	Difference with AN
BOP	118 (2 case control studies)	Low	22.94 (%)	16.57 [12.98, 20.25]
DMFT	244 (4 case control studies)	Low	5.26	2.0 [0.87, -3.13]
Salivary pH	192 (3 case control studies)	Low	6.65	-0.39 [-0.54, -0.24]
Unstimulated salivary flow rate	28 + 38 (4 studies, 2 CCT, 2 case control studies)	Moderate *(due to lack of randomization/ allocation bias)*	0.30	-0.27 [-0.37, -0.21]
α-amylase	232 (4 case control studies)	Low	77.74%	6.32 [-6.13, 18.79]

Nevertheless, it can be concluded from these observations that oral health is measurably impaired in patients with AN. Anorectic patients should be considered risk patients from an orthodontic point of view, as they are more likely to develop decalcification and white spot disease due to their special oral conditions (increased salivary pH and flow rate, increased plaque index). Even without the use of fixed appliances, anorexic patients have an increased caries experience. Especially during fixed orthodontic treatment, it is more difficult to detect dental caries and maintain oral hygiene because of orthodontic appliances. Recent publications in a meta-analysis showed that the incidence of newly developed dental caries lesions during orthodontic treatment was 45.8% [32]. Our results from the meta-analysis of the DMFT Index indicate that this problem may be exacerbated in patients with anorexia. Therefore, special attention should be given to the education of anorexic patients about prevention of dental caries before starting the orthodontic treatment [33].

Adequate oral hygiene is essential for a healthy, functional and pain-free masticatory system. In this context, daily tooth surface cleaning by the patient is of great importance. However, in some cases, biological factors influence the oral environment in such a way that average cleaning is not sufficient to maintain oral health. Such factors may include the presence of toxins of pathogenic bacteria, hormonal disturbances or changes in the amount and/or in the composition of saliva [34]. It is also known that the use of fixed braces as part of orthodontic therapy makes it more difficult for patients to clean their teeth [35]. The combination of biological factors and complications caused by orthodontic therapy can lead to a massive deterioration in a patient’s oral health and irreparable damage to the teeth and periodontium [36]. For this reason, early identification of patients with special biological conditions and a targeted therapy are of paramount importance for successful orthodontic treatment. Specifically, from an orthodontic point of view, patients with AN should be considered as risk patients due to conspicuous oral health. Their oral hygiene should be optimized prior to the initial phase of orthodontic treatment, well monitored and supported during treatment to reduce the risk of caries and periodontitis. Furthermore, interventions should be as short as possible and removable appliances should be preferred to reduce the risk of plaque accumulation and decalcifications.

Saliva has an essential role in the occurrence and progression of dental caries like removal of food debris and sugars, aggregation and elimination of micro-organisms, buffering action to neutralize acid and maintaining supersaturation with respect to tooth mineral composition [37]. Hyposalivation can lead to mucosal lesions which were found to be common in patients with eating disorders [38, 41]. The results in Table 4 support the general assumption that salivary flow rate is reduced in anorexia nervosa. There are several possible explanations for the negative effect of anorexia on parotid gland output, such as dehydration, malnutrition, malabsorption, anemia, hormonal changes and fear [30, 42, 43]. Salivary flow is controlled by the autonomic nervous system [19]. It is also influenced by other factors, such as circulating hormones. Endocrine abnormalities are common in anorexic patients and may result in negatively affected salivation [44]. 

In addition to the quantity, the pH value of saliva is also lowered in patients with AN (Fig. 4). This effect may be caused by acid digestion or fermentation and leads to an enhanced tooth erosion [45]. The buffering and re-mineralizing capacity of saliva may be reduced, making teeth more susceptible to acid attack [18] and the growth of aciduric cariogenic bacteria can be promoted [46]. 

The results of salivary α-amylase concentration showed a high inter-individual variability with no significant evidence. It has been suggested that α-amylase levels may be higher in anorexic and bulimic patients [47]. Studies on the release of α-amylase have shown its dysregulation under conditions of psychological stress [24]. Because of that, salivary α-amylase has been proposed as a reliable marker for the activity of the central component in the automatic nervous system [48, 49]. However, some recent studies provided evidence of overall no effect on the activity of the sympathetic branch of the autonomic nervous system in the acute phase of AN [19, 23]. 

Regarding the type of disease, it has been suggested that women with anorexia nervosa suffer from several dental erosions due to self-induced vomiting. Frequent vomiting per day results in greater contamination of the tooth surface with gastric acid, which means a higher risk of demineralisation. This leads to higher dentine hypersensivity, dental erosion and caries [4]. In addition, reduced tooth stiffness due to various fillings or erosion means less suitable surface for adequate bonding of orthodontic braces using the acid etching technique. The present studies [4, 15] showed a significant prevalence of dental erosion in anorexic patients compared to control groups. However, the Basic Erosive Wear Examination (BEWE) score of 5.2 ± 4.5 [4] and the percentage of 16.2% on the BEWE score of 3–8 [15] indicated a low risk of erosive tooth wear staging. Nevertheless, the presence of multiple dental erosions should lead clinicians to suspect that the patient is suffering from eating disorders. This study must be seen in the light of certain limitations since only few articles with focus on the oral health status of patients with AN could be identified. It is also noticeable that most of the studies included focus mainly on female patients from Europe. It is striking that male patients are also increasingly suffering from anorexia nervosa nowadays. However, the studies on oral manifestations currently only consider the female sex. This may possibly influence the results and lead to a limitation in the generalizability of the results. Furthermore it has to be mentioned that the meta-analysis for the unstimulated saliva flow rate is derived from 5 studies by the same working group. The questions addressed in this study are broad and can only provide an overview of the topic. An orthodontic reference can only be implied, as there is no evidence for orthodontic treatment in anorexia patients.

Based on the systematic information on dentofacial manifestations obtained in this study, the increased oral inflammation in patients with AN should be considered by orthodontists to better choose the type of orthodontic appliances and manage oral hygiene during orthodontic treatment. This is a critical factor influencing the success of planned orthodontic treatment in these patients who are susceptible to gingival inflammation and enamel demineralisation.

## Conclusion

According to currently existing evidence from clinical studies on adolescents and young adult patients with anorexia nervosa, oral health in such patients appears to be negatively affected in multiple parameters compared to healthy patients. The meta-analysis showed that BOP, DMFT, salivary flow rate and pH are significantly altered in patients with anorexia nervosa. The plaque index was higher in the most studies. In contrast, no general relationship between α-amylase levels and anorexia nervosa was detectable. This dataset of general oral health in patients with AN and its meta-analysis in this study is relevant to all areas of clinical dentistry. Patients with AN should also be regarded as high-risk patients in terms of oral health and caries risk due to increased gingival inflammation and reduced salivary flow rate and pH.

## Data Availability

The data underlying this article will be shared on reasonable request to the corresponding author.
